# Clinical Abortions

**Published:** 1858-09

**Authors:** 


### EDITORIAL DEPARTMENT.

---

*Criminal Abortions.*—The periodical medical press, which usually meets the eye of the medical man only, is the sole organ of defence which is used against this terrible crime which is now so rife in what is considered an enlightened and virtuous community, and seems lately to have thrown off even the decency of concealment. Why are we thus left alone to exclaim against this burning outrage? Why is it that the daily press, which assumes to be the guardian of the public welfare, and the religious press, which assumes to be the custodian of the public morals, are silent on this revolting theme? We need not ask the reason why, when we take up any of the daily papers which come into the heart of every family, and are read at the fireside of of every home. They are filled with the iniquitous advertisements of quacks, who, not contented, like our "consumption cures," and scrofula exorcisers, with a traffic in the health of a credulous public, must attack its moral, as well as physical integrity. On us, then, devolves the sole duty of fighting against this fearful evil; and so rapidly is it spreading, that we must awake and do something. Let the matter be brought before our City, County, State, and National Associations! Let our legislatures be memorialized, if we have no laws which can touch these money-thirsty and blood-thirsty scoundrels; and let laws be made so that they may be exterminated, root and branch!

We shall have nothing to say in this article in respect to quacks of an inferior degree: ignorant and money-getting men, who are always as ready to impose upon the public, as the public are ready to be imposed upon. We will always have them in one form or another. The credulity of the masses, and frequently of men of sense and education, is proportioned to their ignorance of a subject. About medicine they know nothing; and are as ready to believe in a "natural bone setter," as in the most eminent surgeon. This we can never be free from; but we *must* be free from quacks whose business is to procure abortion, and whose flaming advertisements are undermining the strongest element of a Christian and enlightened society — female delicacy and purity.

In nearly every newspaper in our land, and even some of a *religious character*, we have advertisements of female pills for producing and regulating the menstrual flow, "*these are never to be taken during the first three months of pregnancy, as they are sure to produce abortion.*" What female could mistake the intention of this advertisement! Few do mistake it, as is shown by the unparalleled success of these nefarious compounds. This, however, is not all; we as physicians, know that there is no absolute certainty of producing abortion by any article of the materia medica; and while these medicines have their success, their reputation suffers from repeated failures. The unblushing effrontery of these knaves has now carried them so far as to actually publish advertisements of INSTRUMENTS *avowedly for the purpose of* "*regulating or limiting offspring without injuring the constitution,*" *vide* the following quoted from a daily paper by the Oglethorpe Med. and Surg. Journal:

"PRIVATE HOSPITAL.—*Ricord's Practice.—Dr. Wm. E. Hoyt's old Established Private Hospital, located in the Arcade, opposite the Post Office, Syracuse, N. Y.*

"Where he will introduce to the notice of those afflicted with any form of private disease, the French System of Cure. This class of complaints he has made a specialty for the last fifteen years, and the knowledge he has of the New Method of Treatment, now in operation in Paris, and the Hospitals of this country, warrants him in saying that none of the forms the disease is wont to assume is without a Sure, Quick and Permanent Remedy. The remedies employed by the Doctor are free from taste or smell, contain no mercury, and require no change of diet, business, or pleasure. Persons can be cured at home by stating their case and addressing Dr. Wm. E. Hoyt. Males or Females who have Spermatorrhœa or Nocturnal Effusion, lose no time, but consult the Doctor and get his Specific, which has saved thousands from the grave, and which is warranted to cure this disease in from three to six weeks, or the money refunded.

"Dr. Hoyt is also agent for Dr. Dumas' Female Monthly Pills. No female should be without them; they can truly be called the 'Female's Friend.'

"A desideratum has been gained in the practice of Medicine hitherto unattained by the medical faculty. Dr. Dumas has used these in his practice, in Paris, (which is the largest of any physician in that city,) for years, and never during thirty years' practice has he been known to have a failure. These Pills have been approved of by the Ecole de Medicine; fully sanctioned by the M. R. C. S., of London, Edinburgh and Dublin, as a never-failing remedy for producing the Catamenial or Monthly flow. Though perfectly harmless to the most delicate, yet ladies are earnestly requested not to mistake their condition, (if pregnant,) as miscarriage would certainly ensue. Price $1.00 per box, sent by mail.

"Dr. Dumas' Female Protecting Instrument, patented and protected by Dr. Dumas, of Paris, and sanctioned by the College of Physicians and Surgeons of London.

"This instrument enables those whose health or circumstances do not permit an increase of family, to regulate or limit offspring without injuring the constitution. The instrument is perfectly safe, no metallic substance entering into its composition; it will last a lifetime without getting out of order, and cannot fail. It can be carried about the person, and used without interference to the conjugal relations of the married state. Those who do not find it as represented, can have the amount of its cost returned. Sent by mail to any part of the U. S. and Canada on the receipt of $5 00. Don't mistake the name or place.

Dr. Wm. E. Hoyt, Syracuse, N. Y."

This is an outrage on which we can no longer be silent; and every man should know and appreciate the blow which is here aimed at the moral sense of a community. Females have now come to regard the production of abortion as one of the most innocent and natural things in the world; and our indignation cannot be unmingled with pity, when we are coolly asked to assist in getting rid of an embryo, with as much "*sang froid*" as we are asked to vaccinate. Our horror and indignation is not in the least understood, and the fair petitioner goes away entirely unconvinced of the nature of the crime which she wishes to commit. Nor is this feeling confined to females in the lower walks of life, or to those unfortunates who are suffering the consequences of the villany of our own sex, who have before them a hopeless gulf of shame, sin and misery. We cannot but compassionate the misfortunes of this latter class, and many a time has the heart of a good man been wrung with the recital of a tale of distress and wrong; and the human feelings have been almost willing to excuse the vile crime which the good physician can never commit. A fair fame blasted; a happy, innocent life changed to a wild and almost unavoidable career of crime and remorse; is a horrible picture to view with firmness. Yet we must look with firmness upon these consequences of frailty, and we must refuse to do the act. which seems alone able to rescue and give back the penitent. There may be an excuse for this; but where is the excuse for those who wish to interrupt the *legitimate* processes of nature. Heretofore it has been thought sufficiently wicked to smother the maternal instincts with the whirl of fashionable gaiety, and to leave one's own flesh and blood to the custody of servants. But now we have ladies, yes, *educated and refined ladies*, who patronize those persons who advertise to prevent an undue increase of family! Who use this instrument which does not interfere "*with the conjugal relations of the married state!*" In charity we must suppose that they know not the crime of which they are guilty. They certainly do not appreciate the extent of disease to which such abuses almost inevitably lead. Nor is it probable that they will ever know it; the daily press utterly ignores the subject, and contents itself with inserting these advertisements, and occasionally calling attention to the card of Dr. So and So, of Paris, "who gives particular attention to the treatment of female diseases."

Let us glance for a moment at the moral wrong of criminal abortion. It is murder; and an unnatural murder which finds no parallel even in the brute creation. The innocent victim, has a circulating, a nervous system, muscles, and all which constitutes life. In a moral point of view there is no difference between the offences of taking the life of a fœtus at three months, and a newly born infant, and even a mature human being; though in the former case the crime is the more heinious from the entire helplessness of the victim. We can sometimes sympathize with the man who has revenged a great wrong by taking the life of a fellow-being. The sudden and uncontrollable passion which sometimes leads to murder, we can explain. Even the necessities of a poor man, or the cupidity of an avaricious one, which prompt the crime of murder, make it excusable in comparison with the killing of helpless infant, which the mere animal instincts of nature, never violated by brutes, though so often annihilated in the human race, teach us to cherish and protect; the deed actuated by an unwillingness to perform those maternal duties, which should contribute so much to the happiness of every virtuous woman.

This picture, which might seem exaggerated to the unprofessional reader, is known by the physician to be plain and truthful; and few there are who do not know of such occurrences, even in the highest walks of society.

Great as this evil is, we think that something can be done to remedy it, and it must be by us that the initiatory step be taken. The matter should be presented to every Medical Association throughout the land, which should appoint a committee of investigation, to consult with the public prosecutors and ascertain of there be at present any means of reaching the difficulty by the law. If there be, let it be tested! If there be not, let the profession, *en masse*, demand of our legislative bodies, a law to prevent this horrible destruction of human life and public morals. A united and powerful movement will effect the end, and the sooner it is done the better. We cannot believe that the proprietors of respectable newspapers, and that respectable druggists who sell the medicines, view the subject in its proper light; and must think that when they do, they will aid us in our endeavors at reformation.

We are confident that editors of medical Journals hold the views which we have expressed, and hope that they will not consider the evil hopeless, but make a strong, a united effort to remedy it. The task will be difficult; but our enlightened age will not suffer the shame of acknowledging that it is impossible.

